# Characteristics of the Genetic Spread of Carbapenem-Resistant *Acinetobacter baumannii* in a Tertiary Greek Hospital

**DOI:** 10.3390/genes15040458

**Published:** 2024-04-05

**Authors:** Martha Papadopoulou, Ioannis Deliolanis, Michalis Polemis, Alkiviadis Vatopoulos, Mina Psichogiou, Panagiota Giakkoupi

**Affiliations:** 1 Laboratory for the Surveillance of Infectious Diseases-LSID, Department of Public Health Policy, University of West Attica, 11521 Athens, Greece; avatopoulos@uniwa.gr (A.V.); pgiakkoupi@uniwa.gr (P.G.); 2 Department of Microbiology, Laiko General Hospital, 11527 Athens, Greece; johndeliol@yahoo.gr; 3 Hellenic National Public Health Organization, 15123 Athens, Greece; m.polemis@eody.gov.gr; 4 1st Department of Internal Medicine, Laiko General Hospital, National and Kapodistrian University of Athens, 11527 Athens, Greece; mpsichog@med.uoa.gr

**Keywords:** *Acinetobacter baumannii*, OXA-type carbapenemases, IS*Aba1*, carbapenem resistance, molecular epidemiology, Greece

## Abstract

*Acinetobacter baumannii* (Ab) has increasingly been identified as a cause of hospital-acquired infections and epidemics. The rise of carbapenem-resistant Acinetobacter baumannii (CRAB) poses significant challenges in treatment. Nosocomial outbreaks linked to CRAΒ *A. baumannii* strains have been reported worldwide, including in Greece. This study aimed to analyze the molecular epidemiology trends of multidrug-resistant *A. baumannii* isolates in a tertiary hospital in Athens, Greece. A total of 43 clinical isolates of extensively drug-resistant (XDRAB), pan-drug-resistant (PDRAB), and CRAB were collected from patients suffering from blood infection, hospitalized between 2016 and 2020 at the internal medicine clinics and the ICU. *A.baumannii* isolates underwent testing for Ambler class B and D carbapenemases and the detection of IS*Aba1*, and were typed, initially, using pulsed-field gel electrophoresis, and, subsequently, using sequence-based typing and multiplex PCR to determine European Clone lineages. The *bla*_OXA-23_ gene accompanied by IS*Aba*1 was prevalent in nearly all *A. baumannii* isolates, except for one carrying *bla*_OXA-58_. The intrinsic *bla*_OXA-51-like_ gene was found in all isolates. No Ambler class B carbapenemases (VIM, NDM) were detected. Isolates were grouped into four PF-clusters and no one-cluster spread was documented, consistent with the absence of outbreak. The study indicated that XDR/PDR-CRAB isolates predominantly produce OXA-23 carbapenemase and belong to European Clone II. Further research is needed to understand the distribution of resistant bacteria and develop effective prevention and control strategies.

## 1. Introduction

*A. baumannii* is an opportunistic nosocomial pathogen and is classified by the World Health Organization as one of the six highly virulent and antibiotic-resistant bacterial ESKAPE pathogens (*Enterococcus faecium*, *Staphylococcus aureus*, *Klebsiella pneumoniae*, *Acinetobacter baumannii*, *Pseudomonas aeruginosa*, and *Enterobacter* spp.) [1,2,3,4]. *Acinetobacter* possesses inherent resistance to desiccants, disinfectants, and essential antimicrobials, greatly aiding in their prolonged persistence and transmission within healthcare settings. A recent comprehensive analysis of the global impact of drug-resistant bacteria in 2019 recognized *A. baumannii* as one of the top six pathogens responsible for antibiotic-resistance-related deaths [5]. 

A key element contributing to the pathogeny of *A. baumannii* is its genetic adaptability, enabling it to rapidly respond to challenging conditions and pressures. This capability to acquire new antibiotic resistance traits and withstand the stresses encountered in hospital environments facilitates the transmission of *A. baumannii* among patients and its persistent presence within healthcare facilities [6]. 

The genomic epidemiology of *A. baumannii* isolates has revealed the worldwide dissemination of distinct clonal lineages, which have spread due to their resistance to various antimicrobials and have been implicated in global epidemics [7]. 

A burning issue is that high-risk lineages of *A. baumannii*, known as international clones (ICs), typically exhibit characteristics of multidrug resistance (MDR), extensive drug resistance (XDR), and carbapenem resistance (CRAB) [8]. Epidemiological research has identified nine international clones (IC1-IC9) of *A. baumannii*, with IC2 being the most prevalent CRAB, frequently occurring endemically and leading to outbreaks [9]. The presence of various IC2 strains and their growing prevalence in clinical settings indicate an ongoing adaptation of this lineage to the hospital environment [10]. Recent findings indicate the emergence of a highly virulent strain of CRAB (theory of evolution), which correlates with elevated mortality rates and clonal spread within hospital settings [11,12].

Carbapenem resistance in *A. baumannii* arises from the interplay of various mechanisms, primarily involving oxacillinases (OXAs) and occasionally metallo-β-lactamases (MBLs) [13]. The overexpression of plasmid- or chromosomally encoded *bla*_OXA_ genes encoding oxacillinases (*bla*_OXA-23_, *bla*_OXA-24/40_, *bla*_OXA-51_, *bla*_OXA-58_, and *bla*_OXA-143_), along with the presence of metallo-carbapenemases (*bla*_IMP_, *bla*_VIM_, *bla_S_*_IM_, and *bla*_NDM_), and the downregulation of porins, which act as channels for carbapenem influx, are common mechanisms [13]. *Bla*_OXA-23_ is preceded by an IS element, IS*Aba1* or IS*Aba4*, and contiguous to a truncated version of ATPase [14,15]. OXAs typically exhibit weak hydrolysis of carbapenems, which theoretically should not lead to resistance development. However, they are sometimes linked with insertion elements that can enhance carbapenemase expression [16,17]. Additionally, the low permeability of the outer membrane in *A. baumannii* also contributes to carbapenem resistance [18]. 

CRAB infections pose significant challenges in healthcare settings due to their antibiotic resistance, elevated mortality rates, and substantial healthcare expenses [19]. CRAB outbreaks have been documented worldwide in hospitals, particularly in intensive care units (ICUs), where they are challenging to contain and can become endemic, highlighting the urgent need to limit their spread from both clinical and public health perspectives. Many of these reports have stressed the importance of comprehensive infection control measures for managing CRAB [20,21]. Identifying carriers is a crucial component in controlling the spread of resistant organisms in healthcare facilities, such as the NG-Test DetecTool OXA-23 assay, which appears to be a reliable, rapid, and cost-effective test suitable for integration into the standard workflow of clinical microbiology labs in regions where OXA-23-producing CRAB infections are prevalent [22]. Early and precise detection enables the implementation of targeted infection control measures, such as isolation protocols and intensified environmental sanitation.

Surveillance data indicate that over 50% of *A. baumannii* isolates from intensive care units (ICUs) in the US and Europe are resistant to carbapenems [23,24].

Greece has been recognized as one of the countries experiencing elevated rates of CRAB infections, especially within hospital environments, and the incidence of CRAB in Greece has been progressively rising over time. According to the European Antimicrobial Resistance Surveillance Network (EARS-Net), carbapenem resistance in *Acinetobacter* species in Greece ranged between 94.8% in 2017 and 96.9% in 2021, and a high prevalence of *Acinetobacter* infections was recorded in Greek hospitals. This suggests that Greece has one of the highest rates (over 80%) of *Acinetobacter* infections in Europe [24,25]. Consistent with these findings, a nine-year study conducted from Southwestern Greece revealed high resistance rates to carbapenems of *A. baumannii* isolated from bloodstream infections (BSIs) and a significant increase in MDR, XDR, or even PDR *Acinetobacter baumannii*-associated nosocomial BSIs [26,27]. Similarly, high levels of carbapenem resistance in *A. baumannii* isolates obtained from blood samples were observed throughout the WHONET Greece data [28]. 

Regarding the molecular epidemiology of clinical CRAΒ isolates in Greece, carbapenemases are the most prevalent mechanism, with *bla*_OXA-23_ being the unique and predominant gene among them [13,29,30]. Since the emergence of the carbapenemase OXA-23 in *Acinetobacter baumannii* in 2010, it has continued to spread throughout Greece, displacing the previously endemic carbapenemase OXA-58. Additionally, it has been observed to exhibit slightly higher minimum inhibitory concentrations (MICs) for carbapenems, attributed to its increased hydrolytic activity [31]. This characteristic has been viewed as a competitive advantage, enabling it to thrive and become predominant in hospital settings. The reported incidence of VIM-producing CRAB, in contrast, was lower, with detection of the *bla*_VIM-1_ and *bla*_VIM-4_ variants [32,33]. A potential explanation for their limited spread could be attributed to the poor carbapenem-hydrolytic activity exhibited by VIM carbapenemases when compared to the OXA-23 and OXA-58 enzymes. Regarding the reported frequency of NDM-producing CRAB, it is increasing rapidly [34], although in Greece it has not been widely documented except for some isolated cases.

Therefore, understanding the epidemiology and the genetic pattern of the organism is essential for the ongoing surveillance of *Acinetobacter* as a crucial component of infection prevention and control efforts.

The present study aimed to investigate the molecular mechanism conferring resistance to carbapenems in *Acinetobacter baumannii* strains isolated as the causative agent of blood infections in patients in ICU and general pathology wards who were hospitalized from 2016 to 2020 in a university tertiary care Greek hospital and to gain further insights into the molecular epidemiology and spread of carbapenem-resistant clinical isolates and genes in order to effectively tackle them.

## 2. Materials and Methods 

### 2.1. Study Design

This retrospective study comprises 43 single-patient clinical isolates of carbapenem-resistant *A. baumannii* (CRAB), obtained from laboratory-confirmed blood infections. These isolates were collected from patients admitted to the General Hospital of Athens “Laiko” between January 2016 and December 2020. Laiko Hospital, an academic tertiary care institution, houses 500 adult beds and accommodates roughly 50,000 admissions annually, serving a population of 2 million residing in the Athens metropolitan region. The hospital comprises several units, including those pertinent to the study: three general medicine wards equipped with both double and triple rooms, along with a 12-bed intensive care unit (ICU). The majority of isolates (37/43, 86.05%) were obtained from patients in the three general medicine clinics, with the remaining 6/43 (13.95%) originating from ICU patients. The sampling process involved selecting the first isolate per month from the pathology clinics and the first isolate per six months from the ICU, as per the Laboratory Informational System. Initially, 61 isolates were identified from the hospital laboratory database, but 17 could not be retrieved from the freezer and 1 isolate was excluded due to misidentification. During the period from April 2020 to November 2020, no samples were stored in the hospital laboratory due to the challenging circumstances and increased workload resulting from the COVID-19 pandemic. This study adhered to the approved guidelines of the Ethics Committee of Laiko General Hospital. The detection of resistance mechanisms was conducted at the Infectious Diseases Surveillance Laboratory, Department of Public Health Policy, West Attica University.

### 2.2. Bacterial Identification and Antimicrobial Susceptibility Testing

Identification and antibiotic susceptibility testing were conducted using the automated system Microscan WalkAway 96 plus (Beckman Coulter, Brea, CA, USA) using a routine laboratory diagnostic process, with minimum inhibitory concentrations (MICs) interpreted according to CLSI guidelines (Clinical & Laboratory Standards Institute) [35]. Due to the lack of CLSI clinical breakpoints, MIC to tigecycline was interpreted according to the recommendation of the US Food and Drug Administration, and susceptibility to tigecycline was determined as MIC ≤ 2 μg/mL [36]. Testing covered nine antimicrobial categories: aminoglycosides (amikacin, gentamicin, tobramycin), carbapenems (imipenem, meropenem), fluoroquinolones (ciprofloxacin, levofloxacin), extended-spectrum cephalosporins (cefepime, cefotaxime, ceftazidime), folate pathway inhibitors (trimethoprim/sulfamethoxazole), penicillin (piperacillin), β-lactam/β-lactamase inhibitor combinations (ampicillin–sulbactam), tetracyclines (tetracycline, tigecycline), and polypeptides (colistin). *A. baumannii* isolates were categorized as multidrug-resistant (MDR—resistant to at least three antimicrobial categories), extensively drug-resistant (XDR—resistant to all antibiotic categories except polymyxin and tigecycline) and pan-drug-resistant (PDR—insensitive to all antimicrobial agents across all classes, as defined by Magiorakos et al. [37]). 

Microbial strains were preserved in cryovials containing skimmed milk and stored in a deep freezer (−70 °C) until further analysis. For laboratory experiments, all microbes were thawed at room temperature, directly recultured on MacConkey and blood agar, and then incubated at 37 °C for 24–48 h.

### 2.3. Detection of Antimicrobial Resistance Mechanisms

Multiplex PCRs were conducted to identify the presence of four primary class D OXA β-lactamase genes (CHDLs)—*bla*_OXA–23-like_, *bla*_OXA–24-like_, *bla*_OXA–51-like_, and *bla*_OXA–58-like_—as well as two class B metallo-β-lactamase genes (*bla*_VIM_ and *bla*_NDM_). Primers and conditions for detecting oxacillinases were prepared following Woodford et al., while those for metalloenzymes were prepared based on ECDC Protocol 7 (laboratory manual for the detection of resistance to carbapenems and colistin) [38,39]. Isolates confirmed positive for oxacillinase genes were further screened for the presence of the IS*Aba1* element upstream of these genes via PCR according to Poirel et al. [13].

### 2.4. Molecular Typing/Pulsed-Field Gel Electrophoresis (PFGE)

Molecular typing using pulsed-field gel electrophoresis (PFGE) was performed in accordance with Seifert et al. [40]. Molecular fingerprints were analyzed using BioNumerics software (Applied Maths, BioMérieux, Marcy-l’Étoile, France) employing the Dice correlation coefficient and the Unweighted Pair Group Method with Arithmetic Mean (UPGMA). Isolates were considered to belong to the same clone if the similarity coefficient was ≥80% [41]. Additionally, two trilocus multiplex PCRs were utilized to selectively amplify Group 1 and Group 2 alleles of *ompA*, *csuE*, and *bla*_OXA-51-like_, facilitating the assignment of sequence groups and major European clones I, II, or III, as described by Turton et al. [42]. Classification of a strain as a member of Group 1 or Group 2 required the amplification of all three fragments in the corresponding multiplex PCR, and the absence of any amplification by the other multiplex PCR. Group 3 isolates were defined by the amplification of only the *ompA* fragment in the Group 2 PCR and the amplification of only the *csuE* and *bla*_OXA-51-like_ fragments in the Group 1 PCR [42].

## 3. Results

### 3.1. Susceptibility of Isolates

**Figure 1:**
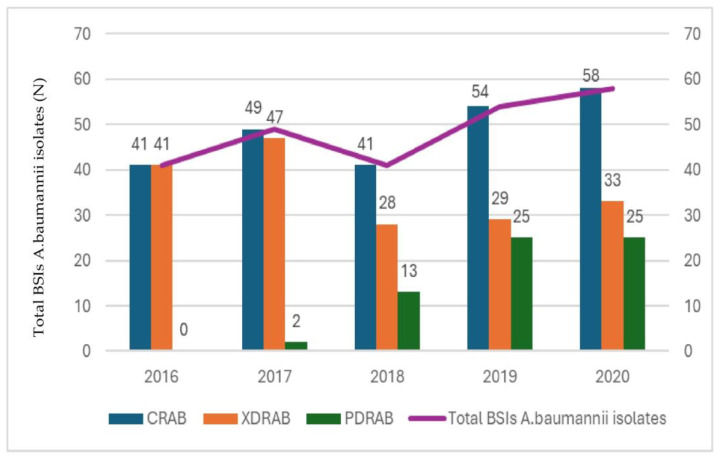
Annual antibiotic resistance of total BSI *A. baumannii* isolates. BSIs: bloodstream infections; CRAB = carbapenem-resistant *A. baumannii*; PDRAB = pan-drug-resistant *A. baumannii*; XDRAB = extensively drug-resistant *A. baumannii*.

To comprehend the antibiotic sensitivity characteristics of the *A. baumannii* strains employed in this research, fifteen antibiotics from nine different classes of antimicrobial agents were tested. All of the blood-isolated A. baumannii strains demonstrated resistance to carbapenems, with the minimum inhibitory concentration (MIC) values for imipenem and meropenem exceeding 16 mg/L. Additionally, these isolates showed resistance to all other classes of antimicrobial agents. The only exceptions were colistin and tigecycline, for which the resistance levels varied. Of the 43 isolates, the resistance rates were 30.2% for colistin and 34.9% for tigecycline, while 83.7% (*n* = 36) were extremely drug-resistant (XDR) and the 16.3% (*n* = 7) were pan-drug-resistant (PDR) in this study. The annual antibiotic resistance rates of both total bloodstream infections (BSIs) and the *A. baumannii* isolates tested in the study are presented in Figure 1 and Table 1, respectively. 

### 3.2. Identification of Class B and D β-Lactamases Genes

The intrinsic *bla*_OXA-51-like_ gene was confirmed in all (100%) isolates. With the exception of one clinical isolate, which tested negative, all others were PCR-positive for the class D OXA β-lactamase gene *bla*_OXA–23-like_. Additionally, 1 out of 43 harbored the β-lactamase *bla*_OXA–58-like_ gene, which was isolated from the first internal medicine clinic in February 2019 and was identified as belonging to a PDRAB strain. None of the isolates contained additional CHDLs, like *bla*_OXA–24-like_. Neither of the class B metallo-β-lactamases genes *bla*_VIM_ or *bla*_NDM_ was detected in any of the isolates.

### 3.3. Mobile Genetic Elements

All isolates except from one with the *bla*_OXA–58-like_ gene were positive for the presence of the insertion sequence IS*Aba1*. The insertion sequence IS*Aba1* was located upstream of the resistant gene in all *bla*_OXA–23-like_ and all *bla*_OXA-51-like_ gene-positive isolates.

### 3.4. Pulsed-Field Gel Electrophoresis (PFGE)

**Figure 2:**
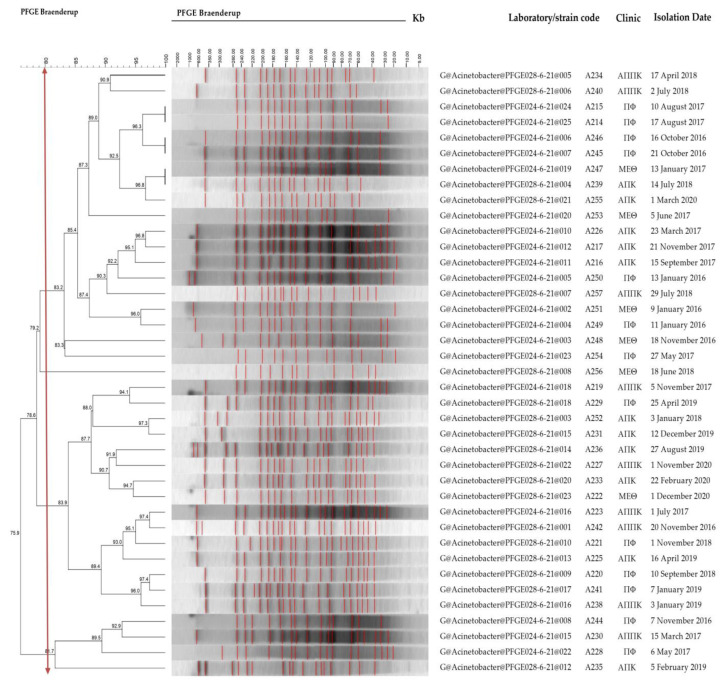
Dendrogram representing the PFGE pattern of 43 clinical *A. baumannii* isolates. Four PF-clusters were identified using a cut-off value of ≥80% for the similarity coefficient (vertical line). The isolation date and clinic from which the samples were obtained are shown.

PFGE was used to determine the genetic and clonal relationships between these clinical isolates using *Apa*I-digested DNA. Molecular typing results demonstrated that the 43 isolates clustered into four groups using a cut-off value of ≥80% similarity (Figure 2). The four pulsed-field clusters of isolates were as follows: PF-Cluster 1, displaying a similarity of 83.2%: 234, 240, 215, 214, 246, 245, 247, 239, 255, 253, 226, 217, 216, 250, 257, 251, 249, 248, and 254, and the 246 strain was further studied; PF-Cluster 2: single isolate 256 and the 256 strain was selected; PF-Cluster 3 displaying a similarity of 83.9%: 219, 229, 252, 231, 236, 227, 233, 222, 223, 242, 221, 225, 220, 241, and 238, and the 227 strain was distinguished; and PF-Cluster 4 displaying a similarity of 81.7%: 244, 230, 228, and 235, and the 235 strain was selected. It is underlined that the 235 isolate possessed *bla*_OXA-58_, differently from the rest of the PF-Cluster 4 isolates possessing *bla*_OXA-23_. The carbapenemase gene could be located on a movable plasmid [43], and not on the chromosome, on which the restriction fragments’ pulsed-field gel electrophoresis was based. This issue was not investigated in the current study.

All clusters were derived from strains isolated from all clinics. We did not observe the presence of a group of indistinguishable subtypes (100% similarity of molecular fingerprints), which would be compatible with an outbreak. The first cluster, which is the most numerous, included strains isolated mainly in the later years, 2016–2017–2018. The third cluster contained the isolates from 2019 to 2020. The fourth cluster included isolates retrieved in 2016–2017–2019.

### 3.5. Multiplex PCR Amplification for ompA, csuE, and bla_OXA-51-like_ Genes

The Trilocus PCR typing of the four representative strains showed that all four belong to Group 1, with the resulting products belonging to Group 1 exclusively: Group 1 *ompA*, sized 355 bp; Group 1 *csuE*, sized 702 bp; and Gp1OXA66, sized 559 bp. Group1 accounts for European clone II. Therefore, all 43 strains of the study belong to the same clade, European Clone II. 

## 4. Discussion

In recent years, the vast majority of *A. baumannii* strains have been resistant to carbapenems and have become the most frequent pathogen in the ICUs in Greek hospitals (http://www.mednet.gr/whonet/ (accessed on 1st January 2024) [44]. It is worth mentioning that during 2015, almost exclusively (>95%) CRAB strains were isolated from Greek hospitals participating in a multicenter study [45]. This observed universal resistance to carbapenems significantly limits active therapeutic options and the interpretation of susceptibility data. The European Antimicrobial Resistance Surveillance Network (EARS-Net) data for 2020, published by the European Center for Disease Prevention and Control (ECDC), reported a very high prevalence of invasive carbapenem-resistant *Acinetobacter* spp. (94.6%) in Greece and a prevalence of over 80% in all Balkan countries [24].

Regarding the development of resistance, it is widely agreed upon that a primary mechanism for carbapenem resistance in *A. baumannii* isolates globally involves the enzymatic alteration or breakdown of β-lactam antibiotics through various β-lactamases, with the most common being OXA-23, OXA-40, and OXA-58 [15,46,47]. 

Screening for OXA-type β-lactamases confirmed the presence of an intrinsic chromosomally located OXA-51-like gene in all the *A. baumannii* isolates in this study. In addition, the OXA-23-like gene was detected in most of the isolates (42/43). All OXA-23-positive strains exhibited resistance to carbapenems and were associated with the presence of an IS*Aba1* element upstream in the gene’s promoter region, potentially enhancing the overexpression of the OXA-23 gene [48,49]. The results of this study align with previous findings indicating that IS*Aba1* promotes the expression of the antimicrobial resistance genes *bla*_OXA-51-like_ and *bla*_OXA-23-like_.

OXA-23 enzymes have become more widespread in recent years and tend to dominate among CRABs worldwide, gradually replacing OXA-58 enzymes [26,50,51,52]. According to published data, CRAΒ isolates collected in Greece in 2015 were primarily associated with International Clone 2 and consistently generated OXA-23, whereas earlier collections before 2004 showed a prevalence of the International Clone 1 lineage and the OXA-58 carbapenemase [29,53,54]. These new strains exhibit increased resistance to carbapenems due to their heightened hydrolytic activity, leading to higher minimum inhibitory concentrations (MICs) [53]. This characteristic has been considered a comparative advantage for survival and predominance in the hospital setting.

While the occurrence of class B β-lactamases is relatively low in Europe, detections of multidrug-resistant strains producing VIM have been reported. A singular strain of CRAB-producing NDM was identified in Greece for the first time in 2016. Recently, there has been a report of the emergence of polyclonal *bla*_NDM-1-positive_ *Acinetobacter baumannii* in a tertiary care hospital in central Greece. [29,34,55]. In our research, VIM and NDM were not found, which is the most likely scenario since they are currently rarely found in Greece.

Molecular characterization reveals that clonal dissemination plays an important role in nosocomial CRAB outbreaks, with most belonging to the international clones 1 (IC1) and 2 (IC2) [56]. The global dissemination of *A. baumannii* resistant to carbapenem antibiotics is recognized as a predominantly clonal phenomenon within hospitals, with IC2 being the most prevalent. A link has been established between strains from the International Clone 2 and isolates that generate OXA-23 [57]. This association is commonly observed among carbapenem-resistant *A. baumannii* strains worldwide. In a previous national study, it was shown that after 2005 the IC2 clone was the most prevalent in Greece compared to the IC1 clone [29,55]. After 15 years, it seems that the IC2 clone continues to dominate in our hospital, as shown by our findings, since according to PFGE and the Trilocus PCR it belongs to European Clone II, which was recently renamed as IC2 [29] and has the common characteristic of producing OXA-23 oxacillinase. The same findings are confirmed by a recent national multicenter study which highlights the dissemination of XDR/PDR *bla*_OXA-23_ harboring *A. baumannii* isolates, corresponding to IC2, in Greek hospitals [58,59]. 

Through core genome phylogenetic analysis, we determined that a single lineage predominantly characterizes our CRAB isolates. PFGE typing did not yield identical molecular fingerprints, indicating no predominant spread of one type in the ICU, likely due to a high level of compliance with hygiene measures and the restriction of spread. The observed clonal relationships in *Acinetobacter* are more likely a result of selection pressure rather than a specific outbreak. Selection pressure could induce the development of virulence attributes and the acquisition of resistance to multiple drugs as part of the adaptation process, thereby aiding the survival of these isolates within a clinical environment. Factors such as prolonged hospital stays, invasive medical procedures, and immunocompromised patients contribute to the transmission and persistence of CRAB.

There are very few antimicrobial agents available on the market that maintain effectiveness against CRAB, such as polymyxins (colistin), aminoglycosides, and tetracyclines (such as tigecycline). However, their utility is limited by suboptimal pharmacokinetic characteristics, and the emergence of resistance and toxicity [60]. Regarding colistin, it is the preferred treatment for severe infections, particularly in the ICU, due to the widespread prevalence of carbapenem-resistant *A. baumannii* strains, which significantly limits treatment options. It is noteworthy that Greece ranked first in hospital colistin consumption among European countries from 2011 to 2015, attributed to the high prevalence of carbapenem-resistant pathogens in Greek hospitals [24]. Consequently, this increased usage has led to a rising trend in colistin resistance, both nationally and globally [24,61]. Colistin resistance poses a greater risk of excess patient mortality [62,63]. To this day, Greece remains the highest consumer of carbapenems and polymyxins within the Europe region [64]. This trend notably corresponds with the observed increase in resistance rates. Specifically, the first study to highlight the simultaneous emergence of carbapenem-resistant *A. baumannii* strains, carrying the blaOXA-23 gene, along with resistance to tigecycline, was conducted in Greek hospitals from 2011 to 2013. This study revealed that all carbapenem-resistant strains belonged to the international CC2 clone [65]. The prevalence of colistin-resistant (ColR) CRAB isolates belonging to IC2 and expressing OXA-23 is increasing, and it represents a huge threat within clinical settings given the very limited effective agents for the treatment of infections caused by such strains [59].

The remarkable resistance rates noted for colistin in this study could be linked to the origin of the isolates (blood) and the prevalence of IC II, as both these factors generally exhibited more resistant profiles compared to non-blood and IC I isolates in a previous national collection of carbapenem-resistant *A. baumannii* strains isolated in 2015 [66]. This trend was also observed in a more recent study [58]. The elevated colistin resistance rate could be attributed to increased colistin usage in Greece, driven by limited therapeutic options against *A. baumannii* [67].

Most CRAB isolates are susceptible to only one or two agents, rendering them extensively drug-resistant (XDR) pathogens [68]. The emergence and dissemination of XDR/PDR *A. baumannii* strains within hospital settings has contributed to elevated mortality rates and presents significant challenges for eradication, emphasizing the urgent need for new and innovative antibiotic treatments [69,70]. In a recent study, there was a significantly higher occurrence of biofilm production and the concurrent presence of *ompA* and other relevant genes among MDR and XDR *A. baumannii* isolates compared to non-XDR-MDR isolates [71]. There is an increasing number of reports regarding Gram-negative bacteria (GNB) exhibiting resistance to carbapenems, aminoglycosides, polymyxins, and tigecycline simultaneously, termed CAPT-resistant strains, including *Acinetobacter baumannii*. Consequently, PDRAB is increasingly reported worldwide [72].

A complete investigation into the reasons behind *A. baumannii*’s continued prevalence in Greek hospitals has not been conducted. However, it is likely that it is linked to the organism’s intrinsic resistance to various antibiotics. Moreover, its ability to survive on surfaces for long periods, combined with the rapid emergence of new resistances and its spread through clones, may also contribute to its persistence [7].

These data underscore the continued significance of XDR/PDR-CRAΒ as a healthcare-associated pathogen with limited treatment options. CRAΒ isolates causing infections in Greek hospitals almost exclusively produce OXA-23 and mainly belong to IC2.

The strengths of the present study are (a) the extensive duration of observation (5 years) and (b) the representative population of patients included in the study, from the University Hospital Laiko of Athens, one of the largest tertiary hospitals in the capital. All these data enable us to draw safe conclusions.

The limitations of the current study include its single-center design, which means that its findings may not be representative of the broader national situation. Additionally, this study encompasses isolates collected from 2016 to 2020, which may not accurately reflect the current epidemiological situation, especially after the SARS-CoV-2 pandemic.

Efforts to develop new antimicrobials must include strategies to address the presence of these enzymes, such as the introduction of new inhibitors to be used in combination with antibacterial drugs. Despite the daunting prospect of facing strains resistant to all available treatments, ongoing research efforts to introduce new inhibitors and drugs offer hope that the options for controlling these infections will persist.

Awareness of the current epidemiological status is crucial in combating the spread of multidrug-resistant *A. baumannii*. Enacting a national surveillance strategy and establishing a rapid carrier detection and isolation mechanism to identify epidemic clusters and notable occurrences within healthcare facilities are indicative of quality care. Maintaining proper infection control measures such as hand hygiene, sterilization, isolation protocols, efficient antimicrobial stewardship, spread control measures, and appropriate antibiotic use mitigates the transmission of infectious diseases within hospitals.

## Figures and Tables

**Table 1:** Antibiotic resistance of tested BSI *A. baumanni* isolates per year.

	2016	2017	2018	2019	2020
Tigecycline resistant	50%	20%	62.5%	37.5%	75%
Colistin resistant	12.5%	6.66%	62.5%	37.5%	25%
XDRAB	100%	100%	93%	97.7%	93%
PDRAB	0	0	7%	2.3%	7%

BSIs: bloodstream infections; PDRAB = pan-drug-resistant *A. baumannii*; XDRAB = extensively drug-resistant *A. baumannii.*

## Data Availability

The data used to support the findings of this study are included within the article, and any additional data are available upon request.
